# Is good more alike than bad? Positive-negative asymmetry in the differentiation between options. A study on the evaluation of fictitious political profiles

**DOI:** 10.3389/fpsyg.2022.923027

**Published:** 2022-07-28

**Authors:** Magdalena Jablonska, Andrzej Falkowski, Robert Mackiewicz

**Affiliations:** Department of Psychology, SWPS University of Social Sciences and Humanities, Warsaw, Poland

**Keywords:** positive-negative asymmetry, positive-negative asymmetry in social discrimination, similarity judgments, object differentiation, candidate evaluation, negativity effect

## Abstract

Our research focuses on the perception of difference in the evaluations of positive and negative options. The literature provides evidence for two opposite effects: on the one hand, negative objects are said to be more differentiated (e.g., density hypothesis), on the other, people are shown to see greater differences between positive options (e.g., liking-breeds-differentiation principle). In our study, we investigated the perception of difference between fictitious political candidates, hypothesizing greater differences among the evaluations of favorable candidates. Additionally, we analyzed how positive and negative information affect candidate evaluation, predicting further asymmetries. In three experiments, participants evaluated various candidate profiles presented in a numeric and narrative manner. The evaluation tasks were designed as individual or joint assessments. In all three studies, we found more differentiation between positive than negative options. Our research suggests that after exceeding a certain, relatively small level of negativity, people do not see any further increase in negativity. The increase in positivity, on the other hand, is more gradual, with greater differentiation among positive options. Our findings are discussed in light of cognitive-experiential self-theory and density hypothesis.

## Introduction

### Positive-negative asymmetry and negative differentiation

People constantly make positive and negative evaluations of others they encounter (Bargh et al., 1992). However, the categories of “good” and “bad” are not symmetrical. Typically, people approach other people and neutral objects in a positive manner (Sears, 1983; Willis and Todorov, 2006; Hoorens, 2014). Still, negative features have been repeatedly found to be more potent, intensive, dominant and informative (for review Baumeister et al., 2001; Rozin and Royzman, 2001). The effect is known as positive-negative asymmetry or valence asymmetry.

Negative entities are also said to be more differentiated, with more complex conceptual representations and a wider response repertoire (Rozin and Royzman, 2001). For instance, studies on word frequencies have shown that whereas positive words appear in written and spoken language more often (Matlin and Stang, 1978), the repertoire of words used to describe negative events and states is more diverse and varied (Rozin et al., 2010). The observation is addressed in density hypothesis, according to which a piece of positive information is more similar to another piece of positive information (and thus the set of positive information is denser), while the structure of negative information is more differentiated (Unkelbach et al., 2008a). The hypothesis was corroborated in studies using spatial arrangement methods, which found positive words to be more closely related to each other compared to negative words (Koch et al., 2016). Likewise experimental research showed not only shorter latency time for the correct categorization of positive words as positive (Unkelbach et al., 2008a) but also their lower discriminability rates in recognition memory tasks (Alves et al., 2015).

A greater differentiation of negative entities is typically attributed to its higher adaptive value. Whereas positive stimuli generally produce approach reactions, an encounter with negative entities requires a more elaborate cognitive appraisal and may lead to diverse behavioral outcomes such as fight/flight/freeze responses (Rozin and Royzman, 2001). Furthermore, studies of economic decision-making show that people react stronger to losses than gains (Tversky and Kahneman, 1991; Bernatzi and Thaler, 1995). For example, they accept higher levels of risk to avoid a loss than to get the gain of the same net value.

### Research gap and study rationale

Although the greater differentiation of negative entities is adaptive for reasons already discussed, it is possible to imagine situations in which the differentiation between positive objects is more beneficial. Apart from loss avoidance, people are also motivated by gain-seeking (Kahneman and Tversky, 1984). Thus, after dismissing all unattractive options, people may be motivated to look for the best alternative from attractive options that are left. These predictions have been supported for example in consumer context in the research on the effect of additional positive and negative features (Dhar and Sherman, 1996; Dhar et al., 1999). It turned out that the participants deferred their choice more if products differed in negative but not positive options, suggesting that positive attributes carried more information.

Similarly, the perception of difference between favorable and unfavorable stimuli has been also studied in social context. For instance, Denrell (2005) hypothesized that the negativity bias may be explained with positive-negative asymmetry in experience sampling. Assuming that information sampling follows a hedonic principle, it may be predicted that an initial negative impression of somebody leads to fewer interactions with that person, whereas a positive appraisal increases the probability of future contacts. Consequently, negative evaluations tend to be more stable, homogeneous and less likely to be changed by new information from further interactions, whereas positive appraisals are a result of numerous interactions and are based on a wider portfolio of features. As a result, the representations of positive entities are more extensive and differentiated. This assumption has been neatly summarized as the liking-breeds-differentiation principle (Alves et al., 2016) and is supported by the research on preference-categorization effect (Smallman et al., 2014; Smallman and Becker, 2017) and the perception of variability among group members (Linville et al., 1989; Rubin and Badea, 2012) which both show a better differentiation for desirable and familiar categories and objects.

Greater differences between negative and not positive options have been proposed in already mentioned density hypothesis (Unkelbach et al., 2008a) which has been empirically tested across many stimuli—including evaluative words, traits, facial features and pictures (Unkelbach et al., 2008b; Unkelbach, 2012; Bruckmüller and Abele, 2013; Alves et al., 2016; Koch et al., 2016). The apparent greater differentiation of positive features has been attributed to the confusion of information valence with content diversity. People do have more information about things they like than the ones they dislike. However, the representations of attractive objects consist predominantly of positive features and those are less diverse than negative features. As a result, disliked options can be perceived as rather different, whereas various liked options will appear similar (Alves et al., 2016). The authors explain this effect with an asymmetrical distribution of positive and negative qualities, so that the range for positivity (regarded as a “norm” and an optimal state) is narrower that the range for negativity (which can have two deviations, i.e., “not enough” and “too much” of a particular dimension).

Drawing on these two contradictory findings, we wanted to investigate how differentiated are the evaluations of differently valenced descriptions of political candidates. We decided to concentrate on politicians because we believe that the evaluation of political candidates is a process that most people at least occasionally undertake and the results of such evaluations have important consequences on the functioning of states and the lives of others. Furthermore, although in the conditions of minimal information people tend to approach others in a positive manner, giving them a benefit of a doubt (Sears, 1983; Willis and Todorov, 2006), this person positivity bias may not necessarily hold for politicians who are generally distrusted by the public (Fiske and Durante, 2014). Thus, the study on political profiles may add interesting insights into the wider and already well-researched field of person perception and differentiation between differently valenced social stimuli. Finally, political candidates are often presented in a form of comparison lists or rankings what fits well with our research design.

The research on person perception often lacks precision found in the research on economic decision-making. For example, it is relatively easy to compare changes in objective values such as variations in price with subjective evaluations of the magnitude of these changes such as estimates of how expensive something is. In social context, such objective measures are more difficult to find. Still, they are necessary if one wants to compare how well people differentiate between positive and negative options. Thus, in our research we propose a numeric and a narrative presentation of candidate information, both of which can be quantified. Apart from other dimensions, we also use similarity to an ideal and bad politician as measures of candidate evaluation. We believe that these two categories create natural comparison standards for candidates running in the elections and can be used to measure how far candidate profiles are on the positivity (similarity to an ideal politician) and negativity (similarity to a bad politician) dimensions.

### Hypotheses and research design

In three experiments, we will investigate the differences in the evaluations of various favorable and unfavorable candidate profiles. According to the negativity effect and density hypothesis, there should be greater differences between a bad and a worse candidate due to a greater prominence and informativeness of negative features. On the other hand, if all available alternatives are unappealing, it may not really matter which of them is worse. After all, all of them are *equally* bad (or at least seem to be). If, on the other hand, the assessment pertains to attractive options, then the appraisal of which of them is better gains on importance. Thus, we predict that a political candidate who has a high number of good features will be evaluated as better than the one with an average number of good features. Whereas, a candidate with an average number of bad features will be evaluated as badly as the one with a high number of bad features. This prediction can be formulated as:

**Hypothesis 1**: There will be greater differences between a favorable and a more favorable candidate profile than between an unfavorable and a more unfavorable one.

Our second goal is to investigate the effect of additional positive and negative information on the evaluation of political candidates. Although overall negative features have been found to be stronger than their positive counterparts (for review see Baumeister et al., 2001; Rozin and Royzman, 2001), the effect of additional features is contextual and dependent on the valence of an object to which these features are added. For instance, a rumor about an alleged affair is likely to tarnish the image of a good politician but will do less harm to a politician whose public perception is already bad. The observation can be explained with such psychological effects as the ratio-difference principle (Stevens, 1957; Kahneman and Tversky, 1979; Gescheider, 1997), figure-ground hypothesis (Lau, 1985; Craig and Rippere, 2014) or contrast effects (Hovland et al., 1957; Bless and Schwarz, 2010), according to which a less frequent feature will stand out and lead to greater changes compared to a more frequent one. Based on this rationale, we predict that additional negative information will be more harmful to favorable candidates (than the unfavorable ones), whereas positive features will increase the evaluation of unfavorable candidate profiles (more than the good ones).

**Hypothesis 2**: Additional positive features (or an increase in feature positivity) will improve the evaluation of an unfavorable candidate profile more than the favorable one, whereas additional negative features (or an increase in feature negativity) will decrease the evaluation of a favorable candidate profile more than the unfavorable one.

The hypothesis assumes a symmetrical effect of additional positive and negative features, following the normative predictions of the contrast model of similarity (Tversky, 1977) which describes similarity between two objects based on the ratio of common and distinctive features. According to the model, a political candidate can be regarded as a set of positive and negative features that describe him or her. Depending on the favorability of candidate image (i.e., the ratio of positive and negative features), additional favorable and unfavorable pieces of information lead to divergent changes in evaluations (Falkowski and Jabłońska, 2018; Falkowski et al., 2021). For instance, although positive features will make all candidates more attractive, the improvement will be more visible in the case of bad candidates than the already good ones.

Still, the model is valence-insensitive and does not account for positive-negative asymmetry. According to normative predictions, two positive features added to an object represented by two positive and four negative features should lead to comparable changes as those produced by two negative features added to an object characterized by two negative and four positive features. However, as shown by Falkowski and Jabłońska (2018), the empirical results tend to diverge from these normative predictions.

Assuming that two additional positive and negative features have the same strength but different valence, two effects are possible. On the one hand, it can be predicted that negative features added to unfavorable candidate profiles will lead to greater changes due to the negativity bias. On the other hand, assuming that people see greater differences among liked than disliked objects, it is reasonable to assume that positive features added to already favorable candidate profiles would still be able to produce a visible increase in candidate evaluation. Thus, joining together two earlier hypotheses, in Hypothesis 3 we want to test the following:

**Hypothesis 3**: Additional negative features will not degrade an image of a unfavorable politician, whereas additional positive features will improve an image of a favorable politician.

The predictions will be tested in three experimental studies. In Study 1 and Study 2 we will present the features of candidates as a set of dimensions (numeric presentation), whereas in Study 3 we will use narrative descriptions of candidates. Additionally, in Study 1 and 3 different participants will evaluate different candidates, whereas in Study 2 participants will be presented with pairs of candidate profiles and asked about the extent of similarity between them. Participants will evaluate candidate profiles with regard to such aspects as their similarity to an ideal and bad politician, liking and voting intention. However, as shown by research on framing and priming effects, the activation of positively and negatively valenced categories (such an image of an ideal or extremely bad politician) may change the appraisal of later presented objects (Kahneman and Tversky, 1984; Weaver, 2007; Bizer and Petty, 2012). Accounting for these effects, we decided to use the valence of the reference point (either ideal or extremely bad politician) as one of the independent variables. All studies were conducted in compliance with APA ethical guidelines (American Psychological Association, 2010) and were approved by the ethical committee of the university at which the research was conducted.

## Study 1

### Materials and methods

#### Participants

Our *a priori* power analysis with the assumption of a medium effect size, an α error probability of 0.05, and a power of 0.80 indicated a required sample size of 34 participants (Faul et al., 2007). Forty two participants took part in our experiment. The sample (72.3% female) was relatively young (*M* = 25.10; *SD* = 8.378) and was not remunerated for participation. On average, participants were slightly disinterested in politics (measured with a 11-point Likert scale, with 0 *not at all interested in politics* and 10 *extremely interested in politics*, *M* = 3.86, *SD* = 2.619) and were neither extremely left- or right-wing oriented (measured with a 11-point Likert scale, with 0 *extreme left* and 10 *extreme right M* = 4.55, *SD* = 1.310).

#### Procedure

The group was randomly divided into two research conditions depending on whether participants were to assess candidates’ similarity to either an ideal or bad politician. The division was introduced to account for potential framing effects, where the activation of certain categories (e.g., an ideal or bad object) serves as a natural reference point and may influence later evaluations of other objects (see Tversky and Kahneman, 1981; Barker, 2005). At the beginning of the experiment, participants were instructed that they would be presented with profiles of five political candidates that ran in parliamentary elections. They were also informed that each candidate would be described along six different dimensions (such as competence and honesty) and that each dimension could take the values of –10 to + 10, where the higher positive value symbolized the greater extent to which a politician possessed a certain positive feature of a particular dimension. Participants were asked to study each candidate profile individually and evaluate it. Each profile was presented separately and the order of the presentation was randomized. Participants could not return to their previous answers. After filling in demographic information, participants were debriefed.

#### Materials

The participants were presented with five candidate profiles that differed in the extent to which they possessed features relevant for a political post. Based on literature review, such aspects as education, qualifications, resourcefulness, honesty, justice and truthfulness were selected (Kinder et al., 1980; Cwalina et al., 2005; Cwalina and Falkowski, 2006). The characteristics were found to be the most common criteria in candidate evaluation. Each of the characteristics was presented on a 21 point bipolar scale, so that zero constituted the neutral point, negative values pertained to the negativity of a feature and positive values to its positivity (e.g., for intelligence, –10 signified *very low intelligence* and + 10 *very high intelligence*). Apart from a neutral candidate whose combined sum of measures on each of the scales equaled 0, there were two negative and two positive candidate profiles. Positive candidate profiles were constructed in such a way that they had either 24 or 48 points toward the positive dimension, whereas negative profiles had either 24 or 48 points toward the negative dimension. We used the same descriptions in Study 2. In what follows we used the following notation to refer to the candidates presented in Study 1 and 2:

- Candidate –48: the sum of scores on all dimensions equals minus 48
- Candidate –24: the sum of scores on all dimensions equals minus 24
- Candidate 0: the sum of scores on all dimensions equals 0
- Candidate + 24: the sum of scores on all dimensions equals plus 24
- Candidate + 48: the sum of scores on all dimensions equals plus 48

Table 1 illustrates the overall valence of candidate profiles used in Study 1. For participants, each candidate was presented individually in a tabular form.

**TABLE 1:** Candidate profiles used in study 1.

	Candidate –48	Candidate –24	Candidate 0	Candidate + 24	Candidate + 48
Resourcefulness	–6	–2	2	2	6
Education	–10	–6	–2	6	10
Qualifications	–8	–4	0	4	8
Honesty	–10	–6	–2	6	10
Justice	–7	–3	1	3	7
Truthfulness	–7	–3	1	3	7

The numbers represent the extent to which a candidate possessed particular features on a scale –10 to + 10.

#### Measures

Participants evaluated each candidate individually with regard to their similarity to either an ideal or bad politician (depending on the experimental group). The question read: *On a scale from 0 to 10 how similar is the candidate to an image of an ideal (bad) politician?*, with answers ranging from 0 *very dissimilar* to 10 *very similar*.

## Results and discussion

**FIGURE 1:**
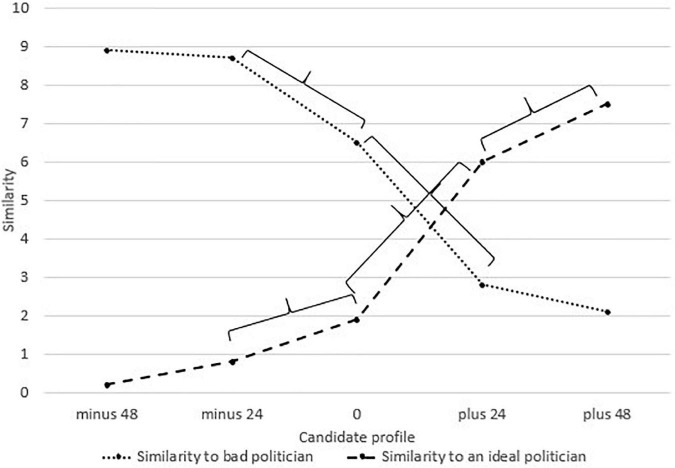
Means of the similarity to an ideal and bad politician for candidates analyzed in study 1. Captions minus 48, minus 24, 0, plus 24, and plus 48 refer to the overall valence of candidate profiles. Brackets mark significant differences (*p* < 0.01) between the analyzed candidates.

Figure 1 presents mean ratings for candidates’ similarity to an ideal and bad politician.

The aim of Study 1 was to test Hypothesis 1 and 3. The initial analysis showed that for most of the variables the data were not normally distributed and exceeded the accepted levels of skewness (Wessa, 2017). Thus, instead of a repeated measures analysis of variance, its non-parametric version, Friedman’s test was used (Bewick et al., 2004) in order to test whether candidate valence (–48, –24, 0 + 24, + 48) differentiated profiles with regard to their similarity to an ideal and bad politician. The results of the analysis were significant for both types of similarity, showing significant differences between candidate profiles with regard to their similarity to an ideal politician, χ^2^(4) = 71.543, *p* < 0.001, and similarity to a bad politician, χ^2^(4) = 59.276, *p* < 0.001.

As the data contained many similar ratings (e.g., zeros as similarity of the worst candidate compared to the ideal politician) instead of traditional parametric or non-parametric testing, permutation tests were used for the *post hoc* checks (see Welch, 1990; Yu, 2002 for the comparison of the approach based on traditional and resampling methods). The probabilities and confidence intervals for re-sampled groups were obtained with the resample package available for R Studio (RStudio Team, 2015). Additionally, we used Cohen’s *d* as a measure of the effect size (Cohen, 1988).

The results for similarity to an ideal politician showed significant differences between candidates –24 and 0 (*p* < 0.001, *d* = –0.895, *CI* = –1.792–0.002), 0 and + 24 (*p* < 0.001, *d* = 2.094, *CI* = –1.03 to 3.158) as well as + 24 and + 48 (*p* < 0.001, *d* = –0.97, *CI* = –1.875 to 0.066). There was no difference between candidates –48 and –24 (*p* = 0.447, *d* = 0.188, *CI* = –0.669 to 1.045). For similarity to an extremely bad politician, there were significant differences between candidates 0 and –24 (*p* < 0.001, *d* = –0.768, *CI* = –1.655 to 0.118) as well as 0 and + 24 (*p* < 0.001, *d* = –1.9, *CI* = –0.869 to 2.93). No differences between candidates –48 and –24 (*p* = 0.483, *d* = 0.164, *CI* = –0.693 to 1.021) as well as + 24 and + 48 (*p* = 0.171, *d* = –0.309, *CI* = –1.169 to 0.552) were found. We show all significant differences in Figure 1.

The analysis of effect sizes suggests greater differences between favorable candidate profiles (i.e., candidates + 24 and + 48, with Cohen’s *d* equal 0.97 for similarity to an ideal politician and 0.309 for similarity to an extremely bad politician) than unfavorable candidates (i.e., candidates –24 and –48, with *d* = 0.188 and *d* = 0.164, respectively), providing evidence for Hypothesis 1. Furthermore, corroborating the predictions of Hypothesis 3, the study showed that an increase in feature negativity did not deteriorate candidate evaluation for candidates who were already perceived as unattractive, whereas an increase in feature positivity improved the evaluation of already favorable candidates. The effect, however, was limited to the similarity to an ideal politician as a dependent variable and was not present in the similarity to a bad politician.

An interesting side note is worth mentioning. The evaluations of unfavorable candidates (–24 and –48) were more extreme, with their similarity to an ideal politician ranging between 0 and 1 and the similarity to a bad politician oscillating between 8 and 9. On the other hand, the evaluations of favorable candidates (+ 24 and + 48) were more moderate, oscillating between 5 and 8 for similarity to an ideal politician and 2 and 3 for similarity to a bad politician (see Figure 1). Thus, it seems that if a candidate is unfavorable, their rejection is the same regardless of the amount of negativity that is connected to them. Also, their evaluation will faster reach the bottom of the scale, whereas the evaluation of favorable candidates will be more gradual and restrained, with even very good candidates not scoring extremely high. Consequently, one will not support candidates whose image is even slightly negative and will pay close attention to those who seem attractive. The observation corroborates the research on greater extremity of negative entities (Rozin and Royzman, 2001) but is also in line with our hypotheses, suggesting more differentiated evaluations of favorable options.

## Study 2

In Study 2, we tested further the perceived differences between favorable and unfavorable candidates. First, we wanted to determine whether the observed differences are also visible in other measures important for candidate evaluation. Thus, in Study 2 we added two new dependent variables: candidate liking and voting intention. Furthermore, although the results of Study 1 showed greater differences between favorable candidate profiles, participants evaluated each candidate individually, so that there was no direct comparison. Thus, in Study 2 we simultaneously presented two candidate profiles (either a favorable candidate and its improved version or an unfavorable one and its deteriorated version) and asked participants to evaluate the perceived difference between them. Again, Hypotheses 1 and 3 were tested.

### Materials and methods

#### Participants

Our *a priori* power analysis with the assumption of a medium effect size, an α error probability of 0.05, and a power of 0.80 indicated a required sample size of 48 participants (Faul et al., 2007). One hundred participants from the same population as those in Study 1 (73% female; *M_*age*_* = 26.76; *SD* = 8.98) took part in the experiment. On average, participants were slightly disinterested in politics (measured as previously, *M* = 3.90, *SD* = 2.615) and were neither extremely left- or right-wing oriented (*M* = 5.26, *SD* = 1.614).

#### Procedure

The procedure was similar to that described in Study 1. This time, however, participants were divided into four research conditions. Two groups were presented with favorable candidate profiles (+ 24, + 48; the same as in Study1), whereas two others with unfavorable ones (–24, –48). From these four groups, two of them were asked to evaluate the similarity to an ideal politician, while the other half answered the question on the similarity to a bad politician. This manipulation was introduced to account for potential framing effects and it pertained only to questions on similarity measures. In all of the groups, participants first evaluated each profile separately with regard to similarity measures, candidate liking and voting intention. The order of the presentation was randomized. In the second part, participants were presented with two candidate profiles simultaneously and asked to evaluate how similar they were.

#### Materials

The same candidate profiles as in Study 1 were used. In the first part of the experiment each candidate profile was presented individually. In the second part, the candidates were presented together. The order of the presentation was counterbalanced.

#### Measures

Similarity measures used in the first part of the experiment were the same as in Study 1. Additionally, participants evaluated each profile with regard to candidate liking (measured with a question *How much do you like the politician?*, with answers ranging from 0 *I dislike the politician a lot* to 10 *I like the politician a lot*) and voting intention (*If the politician ran for an office, how likely are you to vote for him?*, with answers ranging from 0 *I would definitely not vote for the politician* to 10 *I would definitely vote for the politician*). The differentiation between options was measured with one question (*On a scale from 0 to 10 how similar are the two candidates?*) with answers ranging from 0 *very dissimilar* to 10 *very similar*.

## Results and discussion

In order to test Hypothesis 1 and 3, a mixed ANOVA was conducted, with candidate profile (24 vs. 48) as a within factor and candidate valence (positive vs. negative) as a between factor. The results are presented in Table 2.

**TABLE 2:** The means for liking, similarity measures and voting intention of candidate profiles investigated in study 2.

Candidate valence	Positive				Negative			
Candidate profile	+ 24		+48		–24		–48	
	*M*	*SD*	*M*	*SD*	*M*	*SD*	*M*	*SD*
Similarity to an ideal politician	3.88	2.321	7.19	2.191	1.42	1.943	1.38	2.609
Similarity to a bad politician	4.28	2.011	2.36	1.705	7.78	2.315	8.78	2.411
Liking	4.25	2.134	7.20	1.800	1.04	1.443	0.71	1.646
Voting intention	3.65	2.552	7.25	2.199	0.80	1.354	0.82	1.900

Candidates could either be positive (with + 24 or + 48 total score) or negative (with –24 and –48 total score).

For similarity to an ideal politician, there were significant main effects of candidate profile [*F*(1, 50) = 34.307, *p* < 0.001, *eta*^2^ = 0.407] and valence [*F*(1, 50) = 53.174, *p* < 0.001, *eta*^2^ = 0.515] as well as a significant interaction [*F*(1, 50) = 35.940, *p* < 0.001, *eta*^2^ = 0.418]. As shown Table 2, the interaction was largely due to considerably greater differences in the evaluations of positive candidates [with a significant simple effect, *F*(1, 50) = 70.237, *p* < 0.001, *eta*^2^ = 0.584] than negative profiles (*ns*).

For similarity to a bad politician, the main effect of candidate profile was non-significant [*F*(1, 46) = 1.566, *p* = 0.217, *eta*^2^ = 0.033], whereas there were significant valence [*F*(1, 46) = 102.923 *p* < 0.001, *eta*^2^ = 0.691] and interaction effects [*F*(1, 46) = 15.775, *p* < 0.001, *eta*^2^ = 0.255]. Again, simple effects showed much greater differences between positive candidate profiles, [*F*(1, 46) = 14.234, *p* < 0.001, *eta*^2^ = 0.236] than the negative ones (*ns*).

For candidate liking, there were significant main effects of both candidate profile [*F*(1, 98) = 79.878, *p* < 0.001, *eta*^2^ = 0.449] and valence [*F*(1, 98) = 223.594, *p* < 0.001, *eta*^2^ = 0.695] as well as a significant interaction [*F*(1, 98) = 124.764, *p* < 0.001, *eta*^2^ = 0.560]. Again, the interaction was largely due to considerably greater differences in the evaluations of positive candidates [with a significant simple effect, *F*(1, 98) = 206.276, *p* < 0.001, *eta*^2^ = 0.678] than negative profiles (*ns*).

The same pattern of results was also found for voting intention, with significant main effects of both candidate profile [*F*(1, 98) = 91.294, *p* < 0.001, *eta*^2^ = 0.482] and valence [*F*(1, 98) 162.059, *p* < 0.001, *eta*^2^ = 0.623] as well as a significant interaction [*F*(1, 98) = 89.252, *p* < 0.001, *eta*^2^ = 0.477]. Again, the interaction effect can be explained with greater differences in voting intention between positive candidates [with a significant simple effect, *F*(1, 98) = 184.225, *p* < 0.001, *eta*^2^ = 0.653] than negative profiles (*ns*).

The findings provided evidence for Hypothesis 3, showing that whereas an increase in feature negativity did not degrade the image of unfavorable candidates, an increase in candidate positivity improved the evaluation of already favorable candidate profiles. Additionally, the results yet again pointed to greater perceived differences between positive than negative candidate profiles stipulated in Hypothesis 1. Still, as in Study 1, the effect can be only inferred from significant and non-significant differences.

In order to directly test Hypothesis 1, we ran a one-way ANOVA with candidate valence as a between factor, and a similarity between two candidate profiles (measured on a 11-point Likert scale) as a dependent variable. The main effect of candidate valence was significant [*F*(1, 98) = 4.023, *p* = 0.048, *eta*^2^ = 0.039], showing negative candidate profiles to be more similar (*M* = 6.24; *SD* = 2.390) than the positive ones (*M* = 5.27; *SD* = 2.450), thus providing a full test of Hypothesis 1.

Overall, the results of Study 2 corroborated previous findings by showing that people see greater differences between positive candidate profiles than the negative ones. Study 2 showed that the effect holds not only for similarity measures but also liking and voting intention. Furthermore, whereas in Study 1 participants evaluated candidates individually, in Study 2 they were also asked to directly compare the two candidates (either two favorable or unfavorable ones) and rate the similarity between them. Again, we found evidence for a better differentiation between positive options than the negative ones.

## Study 3

Study 1 and Study 2 tested the differences in the perception of favorable and unfavorable candidates whose features were presented as a set of dimensions. In Study 3, we wanted to test whether the same effect would hold if candidates were described in a narrative form. In order to do that, we designed narrative descriptions of eight candidates. Those descriptions differed in the number of positive and negative features that characterized them. In the study, we asked each participant to evaluate two candidate profiles. The order of the presentation was randomized and both evaluations were treated as independent. In the first step, we analyzed whether there were visible differences between the profiles of favorable and unfavorable candidates. In the second step of our analyses, we organized candidates in pairs, so that one of the candidates had always either two positive or two negative features more than the other one. By doing that, we were able to test how the same two positive or negative features added to the description of differently valenced candidates influenced their perception. Thus, apart from testing Hypothesis 1 and 3, we also investigated how additional positive and negative information affect the evaluation of differently valenced candidates (Hypothesis 2).

### Materials and methods

#### Participants

One hundred twenty participants, aged 18–54 (*M* = 24.41, *SD* = 5.449) took part in the experiment. As the *a priori* power analysis suggested a required sample of 240 participants, we asked our sample of 120 students to evaluate two candidates, leading to a required number of participants per condition. Furthermore, the conducted *post hoc* power analysis indicated a sufficient power of 0.87 (Faul et al., 2007) of our study. The sample (57% female) was moderately interested in politics (*M* = 4.33, *SD* = 2.696, on a 11-point scale) and was neither extremely left- or right-wing oriented (*M* = 4.42, *SD* = 1.943, on a 11-point Likert scale, as measured on the same scale as in the two previous studies).

#### Procedure

Each participant was asked to read and evaluate the descriptions of two political candidates. Each candidate was evaluated individually and the order of the presentation was randomized.

#### Materials

For the study, we constructed short (three to four sentences long) narrative descriptions of eight political candidates. The profiles mainly enumerated certain positive and negative characteristics a given candidate was supposed to have. The features were based on previous research (Falkowski and Jabłońska, 2018) and were additionally tested in a pilot study to make sure that positive and negative features used to describe candidates differed in their valence but not overall strength. Each participant evaluated two randomly selected candidate profiles and the order of presentation was randomized. One hundred twenty participants provided in total 240 evaluations of candidate profiles which were treated as independent measures due to randomization.

The candidates differed in the proportion of positive and negative features that described them. The number of features characterizing each candidate is presented in the first column of Table 3. The numbers and signs (“ + ” and “–”) present the number of positive and negative features used to described a candidate. The best possible candidate profile had nine positive and two negative features (Candidate 9 + 2–), whereas the worst possible candidate had two positive and nine negative features (Candidate 2 + 9).

**TABLE 3:** The means for liking, similarity measures and voting intention of candidates analyzed in the study.

Candidate profile	Liking		Similarity to an ideal politician		Similarity to a bad politician		Voting intention	
	*M*	*SD*	*M*	*SD*	*M*	*SD*	*M*	*SD*
9 + 2–	6.2	1.584	5.73	2.016	3.53	1.943	6.13	2.224
7 + 2–	5.72	2.153	5.55	2.063	4.03	2.163	5.86	2.356
9 + 4–	4.63	2.341	4.2	2.483	4.97	2.498	4.07	2.728
7 + 4–	4.48	2.204	3.90	2.119	5.00	2.236	3.94	2.065
4 + 7–	3.23	2.432	3.06	2.658	6.06	2.516	3.06	2.620
4 + 9–	2.87	1.978	2.17	1.733	6.93	2.273	2.30	1.968
2 + 7–	2.55	1.901	2.17	1.583	6.62	2.441	2.10	1.896
2 + 9–	2.47	1.961	2.03	2.042	7.53	2.255	1.83	1.967

Column “candidate profile” summarizes the number of positive features (+) and negative features (−) used in their description. The first four candidate profiles have more positive features than negative ones, whereas for the other four the ratio is reversed.

The features were selected based on prior research in which we generated features characteristic for the category of an ideal and an extremely bad politician. We made sure that each favorable candidate had the same seven positive (*cares for citizens, ensures security, competent, good public speaker, stable in beliefs, consistent, ambitious*) and two negative features (*disloyal, greedy*). The 7 + 2– profile was treated as the base profile and it could differ from other favorable candidate profiles by either additional two positive (*well-educated, committed;* 9 + 2–) or negative features (*lacking culture, not keeping election promises;* 7 + 4–). Additionally, there was a profile 9 + 4– that had two additional positive and two negative features more than the base profile (7 + 2–) and which was used for further comparisons. The same rule applied to the unfavorable candidate profiles, with the base profile having seven negative (*quarrelsome, lazy, greedy, populist, despotic, nepotistic, disloyal*) and two positive (*cares for citizens, ensures security*) features. If a candidate possessed two additional positive or negative features, they were the same as for favorable candidates. Again, a 4 + 9– profile had additional two positive and negative features more than the base profile. Candidate descriptions were rather short and mainly consisted of the enumeration of positive and negative features a candidate possessed.

As observed in Study 1 and 2, the symmetry of numerical values used to describe candidate profiles (i.e., –48, –24, 0, + 24, + 48) produced asymmetrical evaluations of their extremity (with candidates –48 and –24 evaluated extremely negative). Thus, in Study 3 apart from controlling for the number of positive and negative features, we wanted to make sure that the features that we used in candidate descriptions are comparable in their intensity. In order to do that, we ran a pilot study in which we determined that the images of favorable and unfavorable candidate (i.e., 7 + 2– vs. 2 + 7–) did not differ when measured in absolute values when it comes to their image favorability (on a scale –10 to + 10), *F*(1, 120) = 3.105, *p* = 0.098, η^2^ = 0.172. Similarly, there were no differences between additional two positive and negative features that we added to base profiles, *F*(1, 120) = 2.04, *p* = 0.173, η^2^ = 0.120.

#### Measures

Each participant evaluated a candidate in relation to four dependent variables. Similarity to an ideal and bad politician, liking and voting intention were measured as in Study 2.

## Results and discussion

Table 3 presents the means for dependent measures tested in Study 3.

In order to test our hypotheses, we ran a two-way ANOVA with candidate valence and candidate profile as independent variables. As the analysis of main effects does not answer our research problems, below we present the results for interaction effects. The interaction effect was significant for all tested dependent variables: for liking, *F*(3, 232) = 4.431, *p* = 0.005, η^2^ = 0.054, for similarity to an ideal politician, *F*(3, 231) = 5.127, *p* = 0.002, η^2^ = 0.062, for similarity to a bad politician, *F*(3, 232) = 3.404, *p* = 0.018, η^2^ = 0.042, and voting intention, *F*(3, 232) = 7.459, *p* < 0.001, η^2^ = 0.088.

In order to determine whether there were greater differences in the perceptions of favorable than unfavorable candidates, we analyzed the simple effects of candidate profile. The simple effects were significant for favorable candidate profiles, pointing to visible differences between candidates with regard to all tested variables: for liking, *F*(3, 232) = 4.835, *p* = 0.003, η^2^ = 0.059, for similarity to an ideal politician, *F*(3, 231) = 5.979, *p* < 0.001, η^2^ = 0.07, for similarity to a bad politician, *F*(3, 232) = 2;978, *p* = 0.032, η^2^ = 0.037, and voting intention, *F*(3, 232) = 7.993, *p* < 0.001, η^2^ = 0.094. None of the simple effects of candidate valence was significant for unfavorable candidate profiles, showing no differences between any of the candidate profiles. Thus, it provides support for Hypothesis 1.

In order to test Hypothesis 2 and 3, we further investigated the simple effects of candidate valence. First, we organized candidate profiles in pairs, so that the other candidate profile had always two additional positive or negative features more than the first one. For instance, in order to test the effect of additional positive features we compared the evaluations of a candidate who had seven positive and two negative features with the one who had two additional positive features (i.e., nine positive and two negative features). Table 4 presents all candidate pairs that were compared to test the effect of additional features—either positive or negative—on candidate perception together with effect sizes. The significance of differences between two compared candidates was determined based on the results of *post-hoc* tests calculated for the simple effects of candidate valence (with significant differences marked with asterisks in Table 4).

**TABLE 4:** Effect sizes for the effect of additional positive and negative information items for candidate pairs analyzed in study 3.

		Liking	Similarity to an ideal politician	Similarity to a bad politician	Voting intention
Additional features	Pair	d	d	d	d
Positive	2 + 9– vs. 4 + 9–	0.203	0.074	–0.265	0.239
	2 + 7– vs. 4 + 7–	0.31	0.404	–0.226	0.418
	7 + 4– vs. 9 + 4–	0.066	0.13	–0.013	0.054
	7 + 2– vs. 9 + 2–	0.255	0.088	–0.243	0.118
Negative	2 + 7– vs. 2 + 9–	–0.041	–0.076	0.388	–0.14
	4 + 7– vs. 4 + 9–	–0.162	–0.394	0.362	–0.326
	7 + 2– vs. 7 + 4–	–0.569*	–0.789*	0.441	–0.866*
	9 + 2-vs. 9 + 4–	0.788*	–0.678*	0.645*	–0.829*

Significant differences in evaluations are marked by an asterisk.

*Marks confidence intervals significant at 95% level.

The results show that whereas additional positive features did not change candidate evaluation in any of the pairs, additional negative features decreased the evaluation of favorable candidates (i.e., candidates who had seven or nine positive features and two or four negative features) as can be visible in significant and large effect sizes. The only exception was the effect of additional negative features on similarity to a bad politician, where no difference between candidates 7 + 2– and 7 + 4– was found. The lack of the effect seems to be, however, an exception to a general rule and fit well the results of Study 1 and 2 where typically no differences between candidate profiles were found for similarity to a bad politician as a dependent variable. Thus, the results generally provided evidence for Hypothesis 2 (although the effect was restricted to positive and not negative features) but refuted the predictions of Hypothesis 3, which anticipated that additional positive features would increase evaluation of already favorable candidates.

Overall, Study 3 again provided evidence for greater differences between favorable candidate profiles than the unfavorable ones. Additionally, the findings corroborated the negativity effect, showing negative features to be stronger than their positive counterparts. However, as predicted, the effect was limited to favorable candidate profiles. Thus, the results suggest that additional negative features can hurt only candidates whose image is favorable but do no harm to politicians whose image is already tainted. Additionally, no effect of positive features was found, showing that additional positive information did not affect candidate evaluation, regardless of candidate image favorability. On the one hand, the finding is in line with ratio difference principle and the contrast effects but on the other it runs against the results of Study 1 and Study 2. This discrepancy between studies may be attributed to differences in candidate presentation. Perhaps, additional two positive adjectives carried less diagnostic information than their negative counterparts. If so, the effect follows the predictions of density hypothesis but it seems to be limited to linguistic attributes and not information presented in a numeric manner.

## General discussion

In our research we analyzed the perceived differences among the sets of favorable and unfavorable options. More specifically, the aim of our studies was to investigate how people see the difference between good and bad political candidates. Certainly, they would vote for the good ones and not vote for the bad, but how do they compare the good candidate to a better one; and the bad to a worse? We looked for the answers to these questions in three experiments. In Study 1, participants compared the similarity of fictitious candidates to the best possible candidate or the worst possible one. We did not provide descriptions of the best and the worst possible and instead asked the participants to imagine such political figures. On the basis of some preliminary research, we chose some positive and some negative features and used them to prepare descriptions of five different candidates: the very bad, the bad, neutral, the good and the very good one. We presented their descriptions in a form of scales with negative and positive anchors. We used the same five descriptions and the same form of presentation in Study 2. This time, however, the participants not only assessed candidates’ similarities to the best and to the worst possible politicians but also estimated the probability of voting and likeability of the candidates as well as were asked to compare two profiles and decide how similar they were. We slightly changed the design in Study 3 in which we used narrative descriptions of the candidates. We conducted our research in the political setting, because candidate evaluation and selection is a process that many people at least occasionally undertake and which has important social, political and economic implications.

Our focus was on the differences between the evaluations of positive and negative candidates. The literature on differentiation provides evidence for two contradictory effects. On the one hand, negative information has been found to have more complex conceptual representations and lead to a wider response repertoire (Rozin and Royzman, 2001). Linguistic research and studies using spatial arrangement methods have also shown negative categories to be more diverse, with more words used to describe negative events and states (Rozin et al., 2010). Likewise, the proponents of density hypothesis (Unkelbach et al., 2008a) found that positive entities are more related (and thus denser) compared to their negative counterparts. On the other hand, literature provides convincing evidence for an opposite effect, that is a better differentiation between positive entities. For instance, Denrell (2005) found that people have more knowledge and more differentiated representations of liked than disliked social stimuli. In a similar vein, Smallman and others (Smallman et al., 2014; Smallman and Becker, 2017) have shown that people make finer evaluative distinctions when rating appealing than unappealing options.

Following this line of research, we assume better differentiation between positive and not negative options to be a norm, especially when making evaluations of social objects or deciding which option to select. Thus, in our research we predicted that participants would be more likely to see the difference between favorable than unfavorable candidates. In our settings that should result in different evaluations of the good and the best candidates, while the evaluations of the bad and the worst one should not differ (Hypothesis 1). We also predicted that additional information about the candidates would be more likely to change a candidate’s image if the valence of the extra information is opposite to the current image. That is, if a candidate is already favorable, the new positive information might help him or her only to some degree, while negative information would significantly harm his or her image. On the contrary, when a candidate is presented in a negative manner, a new piece of negative information would not hurt him or her much, whereas an additional piece of positive information might be quite beneficial for the candidate’s image (Hypothesis 2). Finally, drawing on two earlier hypotheses—on the better differentiation of positive options and an asymmetrical effect of additional positive and negative features—we formulated a hypothesis that joined together these two predictions, assuming that additional positive information would improve the evaluation of an already good candidate, whereas additional negative information would not harm a bad candidate profile (Hypothesis 3).

The results supported our hypotheses. In Study 1 and Study 2 we found that there were no differences in the evaluations of negative candidates, such as a candidate with overall score –24 and a candidate with overall score –48 (the numbers refer to the balance of the evaluations on six different dimensions) were perceived as equally bad. Still, the participants perceived candidates with overall scores + 24 and + 48 as significantly different. The effect was replicated in Study 3, in which candidates were described in a narrative form. This result supports our Hypothesis 1. Importantly, whereas the results of Study 1 and 3 provided only an indirect test of the hypothesized effect, Study 2 gave a direct test as the participants saw both profiles together and were asked to assess their perceived similarity.

Our second research interest was to test how additional positive and negative pieces of information change candidate perception depending candidate valence. As expected, positive features increased candidate evaluation, whereas negative ones decreased it but these effects were not symmetrical, undermining the normative predictions of for instance the contrast model of similarity. This confirms our Hypothesis 2. Furthermore, we obtained a mixed support for Hypothesis 3. The results of Study 1 and Study 2 showed that whereas adding negative features to a candidate’s profile would not change his or her evaluation when this profile was already negative, additional positive features strengthened the image of a unfavorable candidate. However, we did not observe any effect of additional positive features in the evaluations of candidates whose images were presented in a narrative form in Study 3. One possible explanation is that two additional positive characteristics carried less information (i.e., were less diagnostic) than their negative counterparts.

Overall, our findings suggest that people do not see much of a difference between political candidates with many negative features, regardless of the extent to which they are presented as bad. As it seems, at least in the political domain, if an overall evaluation goes below some standard, people do not differentiate between bad options. The effect may be attributed to different motivations in the processing of positive and negative options. If all available alternatives are unappealing, it does not really matter which one of them is worse. After all, they all seem equally bad and, indeed, why anyone would support a bad candidate? This was the case for assessing the similarity to an ideal or bad politician (Study 1, 2, and 3) as well as liking and voting intention (Study 2 and 3). Thus, regardless of their initial expectations people would not vote for a politician if his or her features fall below a certain standard. One possibility that explains this effect is that they would not be able to justify their decision (Shafir et al., 1993).

Importantly, even the standards of “good” and “bad” are not symmetrical, so that it is relatively easy to be deemed as inadequate for the post but rather difficult to be perceived as a good candidate. The effect was especially visible in Study 1 and 2, where there was a dramatic drop in the evaluation of unfavorable candidates, with extremely low, bottom values for candidates’ similarity to an ideal politician and very high similarity to a bad politician. This extremity effect can partially account for the lack of differentiation between negative options. Still, no differences between unfavorable candidate profiles, as predicted in Hypothesis 1, were also found in Study 3, where candidates were presented in a narrative manner and where evaluations were less extreme. Overall, the results of three studies follow our Hypothesis 1, in which we predicted that the evaluations of negative candidates should not differ significantly. However, if the judgment pertains to attractive options, then the decision which one of them is better gains on importance. As visible in our studies, there were significant differences between favorable candidates. Importantly, no ceiling effect was observed. Thus, the bottom effects observed for negative candidate profiles were not paralleled by the symmetrical ceiling effect for positive candidates, suggesting that the participants differentiated their answers when they thought such differentiations were appropriate, providing evidence for better differentiation between positive options.

The results may be explained with regard to two independent information processing systems proposed by Epstein in his cognitive-experiential self-theory (Epstein, 1990; Kirkpatrick and Epstein, 1992). The evolutionally older experiential system operates in an automatic and holistic manner, whereas the rational system is “a deliberative, verbally mediated, primarily conscious analytical system that functions by a person’s understanding of conventionally established rules of logic and evidence” (Denes-Raj and Epstein, 1994, p. 819). It seems that whereas an intense dislike toward negative options is an outcome of the experiential system, a better and more discriminative analysis of positive options is governed by the rational system. The finding can be also interpreted with the distinction into sufficient and necessary conditions, where a necessary condition is one which must be present in order for the event to occur but it does not guarantee the event, while a sufficient condition is a condition that will produce the event. Thus, it seems that the list of necessary conditions to be deemed as inadequate for the post is much shorter than the one for an ideal politician. Consequently, the standards for what it means to be good and bad are not symmetrical.

Our findings have important implications for density hypothesis (Unkelbach et al., 2008a; Alves et al., 2016), according to which the distribution range of positivity is much narrower than the range of negativity. It seems reasonable to assume that an optimal spectrum is narrower than the negative one and, as shown in many empirical studies on density hypothesis, that the inner structure of positive information is denser than the structure of negative entities. Still, in our opinion it does not imply a better differentiation between negative options. As our studies suggest, the structure of positive categories may be denser but this density is accompanied by (or maybe is a reason for) a better discrimination between favorable options. After all, after rejecting all negative alternatives, people put in much effort to decide which of the remaining options is the best or at least acceptable—although the extent of this effort is moderated by decision importance and individual differences (e.g., a distinction into maximisers and satisficers Schwartz et al., 2002). Thus, if the structure of positive entities is denser, it is likely that people use finer combs to disentangle it.

We are aware of some important drawbacks of our study. First, we did not investigate how people estimate real candidates and, consequently, we did not take into account the importance of political views or associations that some voters may feel for different political parties. This research direction should be taken by other scholars. For instance, it is interesting to analyze how well people differentiate between candidates that are from their party compared to the members of the opposing party. Furthermore, the way we constructed our candidate profiles may pose certain limitations on the ecological validity of the study. Although, the use of such profiles was justified by our intention to have a maximal control over analyzed stimuli, further studies should investigate more complex stimuli. Also, it is interesting to analyze how well people differentiate between options, depending on the modality in which they were presented. For instance, in our studies we found that numerical candidate profiles were evaluated more extremely than candidates presented descriptively. Thus, presentation modality as well as the range of a positive and negative spectrum are further areas of research. Overall, our research provides valuable insight into positive-negative asymmetry with regard to a less-explored area of a differentiation between positive and negative options in the political setting. Contrary to the findings on the better differentiation between negative options, we find evidence for the opposite effect, showing that the evaluations of a few favorable objects are actually more nuanced.

## Data availability statement

The raw data supporting the conclusions of this article will be made available by the authors, without undue reservation.

## Ethics statement

The studies involving human participants were reviewed and approved by the Research Ethics Committee at SWPS University of Social Sciences and Humanities, Warsaw, Poland (approval number 12/2020). Written informed consent for participation was not required for this study in accordance with the national legislation and the institutional requirements.

## Author contributions

MJ and AF conceived and planned the experiments. MJ carried out the experiments and took the lead in writing the manuscript. MJ and RM performed the analytic calculations. RM and AF provided critical feedback and helped shape the research, analysis, and manuscript. All authors contributed to the article and approved the submitted version.

## References

[B1] Alves H. Koch A. S. Unkelbach C. (2016). My friends are all alike – the relation between liking and perceived similarity in person perception. *J. Exp. Soc. Psychol.* 62 103–117. 10.1016/j.jesp.2015.10.011

[B2] Alves H. Unkelbach C. Burghardt J. Koch A. S. Krüger T. Becker V. D. (2015). A density explanation of valence asymmetries in recognition memory. *Mem. Cogn.* 43 896–909. 10.3758/s13421-015-0515-5 25772462

[B3] American Psychological Association (2010). *Ethical Principles of Psychologists and Code of Conduct.* Washington, DC: American Psychological Association.

[B4] Bargh J. A. Chaiken S. Govender R. Pratto F. (1992). The generality of the automatic attitude activation effect. *J. Pers. Soc. Psychol.* 62 893–912. 10.1037/0022-3514.62.6.893 1619549

[B5] Barker D. C. (2005). Values, frames, and persuasion in presidential nomination campaigns. *Polit. Behav.* 27 375–394. 10.1007/s11109-005-8145-4

[B6] Baumeister R. F. Bratslavsky E. Finkenauer C. Vohs K. D. (2001). Bad is stronger than good. *Rev. Gen. Psychol.* 5 323–370. 10.1037//1089-2680.5.4.323

[B7] Bernatzi S. Thaler R. H. (1995). Myopic loss aversion and the equity premium puzzle. *Q. J. Econ.* 110 73–92.

[B8] Bewick V. Cheek L. Ball J. (2004). Statistics review 10: nonparametric methods. *Crit. Care* 8:196. 10.1186/cc1820 15153238 PMC468904

[B9] Bizer G. Y. Petty R. E. (2012). How we conceptualize our atiitudes matters?: how we conceptualize our attitudes on the resistance the effects of valence framing of political attitudes. *Polit. Psychol.* 26 553–568. 10.1111/j.1467-9221.2005.00431.x

[B10] Bless H. Schwarz N. (2010). “Mental construal and the emergence of assimilation and contrast effects: the inclusion/exclusion model,” in *Advances in Experimental Social Psychology*, 1st Edn, Vol. 42 ed. Zanna M. P. (Amsterdam: Elsevier Inc). 10.1016/S0065-2601(10)42006-7

[B11] Bruckmüller S. Abele A. E. (2013). The density of the big two: how are agency and communion structurally represented? *Soc. Psychol.* 44 63–74. 10.1027/1864-9335/a000145

[B12] Cohen J. (1988). *Statistical Power Analysis for the Behavioral Sciences.* Mahwah, NJ: Erlbaum. 10.1234/12345678

[B13] Craig S. C. Rippere P. S. (2014). Political trust and negative campaigns: two tests of the figure-ground hypothesis. *Polit. Policy* 42 693–743. 10.1111/polp.12091

[B14] Cwalina W. Falkowski A. (2006). “Political communication and advertising in Poland,” in *The Sage Handbook of Political Advertising*, eds Kaid L. L. Holtz-Bacha C. (Thousand Oaks, CA: SAGE Publications), 325–466.

[B15] Cwalina W. Falkowski A. Kaid L. L. (2005). Advertising and the image of politicians in evolving and established democracies: comparative study of the polish and the US presidential elections in 2000. *J. Polit. Market.* 4 19–44. 10.1300/J199v04n02_02

[B16] Denes-Raj V. Epstein S. (1994). Conflict between intuitive and rational processing: when people behave against their better judgment. *J. Pers. Soc. Psychol.* 66, 819–829. 10.1037//0022-3514.66.5.819 8014830

[B17] Denrell J. (2005). Why most people disapprove of me: experience sampling in impression formation. *Psychol. Rev.* 112 951–978. 10.1037/0033-295X.112.4.951 16262475

[B18] Dhar R. Sherman S. J. (1996). The effect of common and unique features in consumer choice. *J. Consum. Res.* 23 193–203. 10.1086/209477

[B19] Dhar R. Nowlis S. M. Sherman S. J. (1999). Comparison construction effects on preference. *J. Consum. Res.* 26 293–306. 10.1086/209564

[B20] Epstein S. (1990). “Cognitive experiential self theory,” in *Handbook of Personality: Theory and Research*, ed. Pervin L. (New York, NY: Guilford Press), 165–192.

[B21] Falkowski A. Jabłońska M. (2018). Positive–negative asymmetry in the evaluations of political candidates. The role of features of similarity and affect in voter behavior. *Front. Psychol.* 9:213. 10.3389/fpsyg.2018.00213 29535663 PMC5835317

[B22] Falkowski A. Sidoruk-Błach M. Olszewska J. Jabłońska M. (2021). Positive-negative asymmetry in evaluation of natural stimuli: empirical study in the contrast model of similarity extended to open sets. *Am. J. Psychol.* 134 1–11.

[B23] Faul F. Erdfelder E. Lang A.-G. Buchner A. (2007). G*Power 3: a flexible statistical power analysis program for the social, behavioral, and biomedical sciences. *Behav. Res. Methods* 39 175–191. 10.1109/ISIT.2013.6620417 17695343

[B24] Fiske S. T. Durante F. (2014). “Never trust a politicians? Collective distrust, relational accountability, and voter response,” in *Power, Politics, and Paranoia: Why People are Suspicious about Their Leaders*, eds van Prooijen J. W. van Lange P. A. M. (Cambridge, MA: Cambridge University Press), 91–105.

[B25] Gescheider G. A. (1997). *Psychophysics: The Fundamentals.* Mahwah, NJ: Erlbaum.

[B26] Hoorens V. (2014). “Positivity bias,” in *Encyclopedia of Quality of Life and Well-Being Research*, ed. Michalos A. C. (New York, NY: Springer), 4938–4941.

[B27] Hovland C. I. Harvey O. J. Sherif M. (1957). Assimilation and contrast effects in reactions to communication and attitude change. *J. Abnorm. Soc. Psychol.* 55 244–252. 10.1037/h0048480 13474895

[B28] Jabłońska M. Falkowski A. Mackiewicz R. (2022). Is good more alike as bad? Positive-negative asymmetry in the differentiation between options. Study on the perception of political candidates. *Res. Square* [Preprint]. Available online at: 10.21203/rs.3.rs-1193574/v (accessed April 14, 2022). PMC9368193 35967663

[B29] Kahneman D. Tversky A. (1979). Prospect theory: an analysis of decision under risk. *Econ. J. Econ. Soc.* 47 263–291. 10.1111/j.1536-7150.2011.00774.x

[B30] Kahneman D. Tversky A. (1984). Choices, values, and frames. *Am. Psychol.* 39 341–350. 10.1037/0003-066X.39.4.341

[B31] Kinder D. R. Peters M. D. Abelson R. P. Fiske S. T. (1980). Presidential prototypes. *Polit. Behav.* 2 315–337. 10.1007/BF00990172

[B32] Kirkpatrick L. A. Epstein S. (1992). Cognitive-experiential self-theory and subjective probability: further evidence for two conceptual systems. *J. Pers. Soc. Psychol.* 63 534–544. 10.1037//0022-3514.63.4.534 1447684

[B33] Koch A. Alves H. Krüger T. Unkelbach C. (2016). A general valence asymmetry in similarity: good is more alike than bad. *J. Exp. Psychol. Learn. Mem. Cogn.* 42 1171–1192. 10.1037/xlm0000243 26866655

[B34] Lau R. R. (1985). Two explanations for negativity effects in political behavior. *Am. J. Polit. Sci.* 29 119–138. 10.2307/2111215

[B35] Linville P. W. Fischer G. W. Salovey P. (1989). Perceived distributions of the characteristics of in-group and out-group members: empirical evidence and a computer simulation. *J. Pers. Soc. Psychol.* 57 165–188. 10.1037/0022-3514.57.2.165 2760805

[B36] Matlin M. W. Stang J. D. (1978). *The Pollyanna Principle. Selectivity in Language, Memory, and Thought.* New York, NY: Schenkman.

[B37] Rozin P. Royzman E. B. (2001). Negativity bias, negativity dominance, and contagion. *Pers. Soc. Psychol. Rev.* 5 296–320. 10.1207/S15327957PSPR0504_2

[B38] Rozin P. Berman L. Royzman E. B. (2010). Biases in use of positive and negative words across twenty natural languages. *Cogn. Emot.* 24 536–548. 10.1080/02699930902793462

[B39] RStudio Team (2015). *RStudio: Integrated Development for R.* Boston, MA: RStudio, Inc.

[B40] Rubin M. Badea C. (2012). They’re all the same!. But for several different reasons: a review of the multicausal nature of perceived group variability. *Curr. Direct. Psychol. Sci.* 21 367–372. 10.1177/0963721412457363

[B41] Schwartz B. Ward A. Monterosso J. Lyubomirsky S. White K. Lehman D. R. (2002). Maximizing versus satisficing: happiness is a matter of choice. *J. Pers. Soc. Psychol.* 83 1178–1197. 10.1037/0022-3514.83.5.1178 12416921

[B42] Sears D. O. (1983). The person-positivity bias. *J. Pers.* 44 233–250. 10.1037/0022-3514.44.2.233

[B43] Shafir E. Simonson I. Tversky A. (1993). Reason-based choice. *Cognition* 49 11–36. 10.1016/0010-0277(93)90034-S 8287671

[B44] Smallman R. Becker B. (2017). Motivational differences in seeking out evaluative categorization information. *Pers. Soc. Psychol. Bull.* 43 1020–1032. 10.1177/0146167217704191 28903700

[B45] Smallman R. Becker B. Roese N. J. (2014). Preferences for expressing preferences: people prefer finer evaluative distinctions for liked than disliked objects. *J. Exp. Soc. Psychol.* 52 25–31. 10.1016/J.JESP.2013.12.004

[B46] Stevens S. S. (1957). On the psychophysical law. *Psychol. Rev.* 64 153–181. 10.1037/h0046162 13441853

[B47] Tversky A. (1977). Features of similarity. *Psychol. Rev.* 84 327–352. 10.1037/0033-295X.84.4.327

[B48] Tversky A. Kahneman D. (1981). The framing of decisions and the psychology of choice. *Science* 211 453–458. 10.1007/978-1-4613-2391-4_2 7455683

[B49] Tversky A. Kahneman D. (1991). Loss aversion in riskless choice: a reference-dependent model. *Q. J. Econ.* 106 1039–1061. 10.2307/2937956

[B50] Unkelbach C. (2012). Positivity advantages in social information processing. *Soc. Pers. Psychol. Compass* 6 83–94. 10.1111/j.1751-9004.2011.00407.x

[B51] Unkelbach C. Fiedler K. Bayer M. Stegmüller M. Danner D. (2008a). Why positive information is processed faster: the density hypothesis. *J. Pers. Soc. Psychol.* 95 36–49. 10.1037/0022-3514.95.1.36 18605850

[B52] Unkelbach C. Guastella A. J. Forgas J. P. (2008b). Oxytocin selectively facilitates recognition of positive sex and relationship words: short report. *Psychol. Sci.* 19 1092–1094. 10.1111/j.1467-9280.2008.02206.x 19076479

[B53] Weaver D. H. (2007). Thoughts on agenda setting, framing, and priming. *J. Commun.* 57 142–147. 10.1111/j.1460-2466.2006.00333.x

[B54] Welch W. J. (1990). Construction of permutation tests. *J. Am. Stat. Assoc.* 85 693–698. 10.1080/01621459.1990.10474929

[B55] Wessa P. (2017). *Skewness and Kurtosis Test (v1.0.4). In Free Statistics Software (v1.2.1). Office for Research Development and Education.* Available online at: https://www.wessa.net/rwasp_skewness_kurtosis.wasp (accessed February 22, 2022).

[B56] Willis J. Todorov A. (2006). First impressions: making up your mind after a 100-ms exposure to a face. *Psychol. Sci.* 17 592–598. 10.1111/j.1467-9280.2006.01750.x 16866745

[B57] Yu C. H. (2002). Resampling methods: concepts, applications, and justification. *Pract. Assess. Res. Eval.* 8:19.

